# Marketing for Sensa: A novel “zero nicotine vapor product” from a major tobacco company

**DOI:** 10.21203/rs.3.rs-5160841/v2

**Published:** 2024-10-22

**Authors:** Meagan O. Robichaud, Eugene M. Talbot, Ollie Ganz, Melissa Mercincavage, Hanno C. Erythropel, Julie B. Zimmerman, Sairam V. Jabba, Sven E. Jordt, Cristine D. Delnevo

**Affiliations:** Rutgers Institute for Nicotine and Tobacco Studies, New Brunswick, NJ, USA; Rutgers Institute for Nicotine and Tobacco Studies, New Brunswick, NJ, USA; Rutgers Institute for Nicotine and Tobacco Studies, New Brunswick, NJ, USA; Rutgers Institute for Nicotine and Tobacco Studies, New Brunswick, NJ, USA; Department of Chemical and Environmental Engineering, Yale University, New Haven, CT, USA; Department of Chemical and Environmental Engineering, Yale University, New Haven, CT, USA; Department of Anesthesiology, Duke University School of Medicine, Durham, NC, USA; Department of Anesthesiology, Duke University School of Medicine, Durham, NC, USA; Rutgers Institute for Nicotine and Tobacco Studies, New Brunswick, NJ, USA

## Abstract

On July 1, 2024, RJ Reynolds Vapor Company (RJRVC) introduced Sensa, the first disposable “zero nicotine vapor product” from a major tobacco company. Sensa sold more than 100,000 units as of September 10, 2024 and has been marketed through several channels. Direct-mail and magazine ads appeal to consumers’ senses (e.g., “awaken your senses”) and showcase Sensa’s six flavor options: Berry Fusion, Berry Watermelon Fusion, Blueberry Frost, Mint Frost, Passionfruit Frost, and Watermelon Frost. Our chemical characterization of all Sensa flavors (except Berry Watermelon Fusion) found neotame (a potent artificial sweetener) and WS-23 (a synthetic coolant) in all samples, while all “Frost” flavors tested contained another synthetic coolant, WS-3. Although RJRVC states that Sensa is for “adult tobacco and vapor consumers,” Sensa’s resemblance to popular disposable vaping devices and youth-appealing flavors raise concerns about Sensa’s appeal to young people, and limited regulations for “zero nicotine” products introduce uncertainty about product safety.

## Introduction

On July 1, 2024, RJ Reynolds Vapor Company (RJRVC)—an operating company of British American Tobacco’s (BAT’s) subsidiary, Reynolds American Inc.—introduced Sensa, a “zero nicotine” disposable “vapor product,” in the United States (U.S.).^1^ Sensa resembles popular nicotine-containing disposable devices from brands such as Lost Mary, as well as BAT’s own Vuse Go 5000 offered outside the U.S.^2–4^ While several “zero nicotine” devices are available, Sensa is the first “zero nicotine” disposable device from a major tobacco company, perhaps in response to the popularity of disposable vaping devices. Since Sensa contains neither nicotine nor tobacco, RJRVC claims exemption from U.S. Food and Drug Administration regulations.^5^

**Figure 1:**
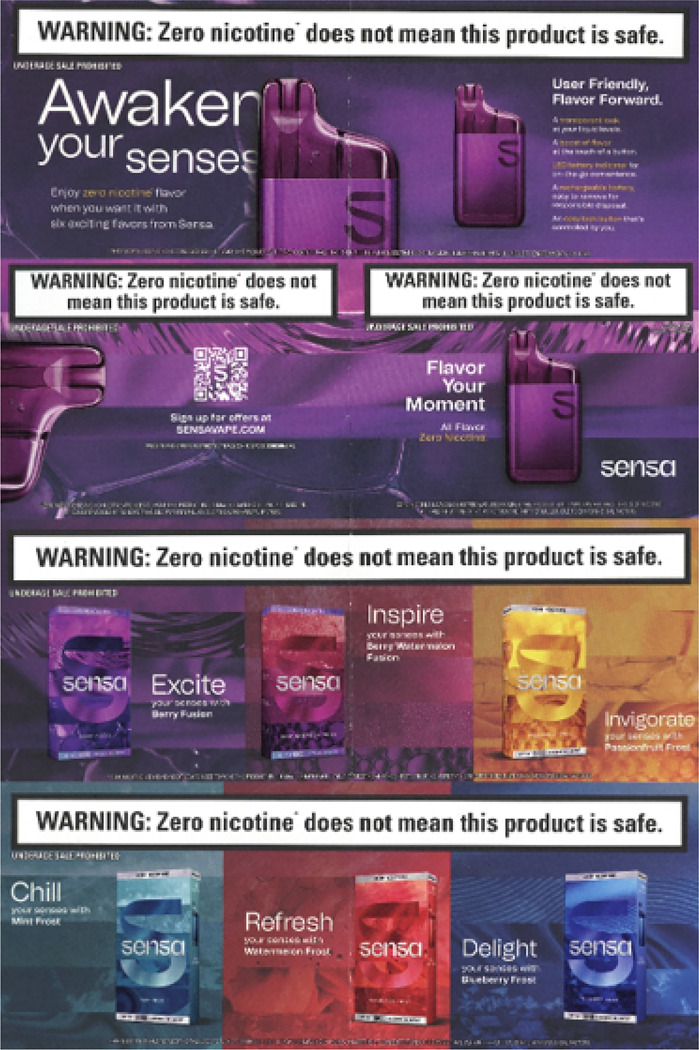
Direct-mail ad for Sensa (received August 2024)

**Figure 2:**
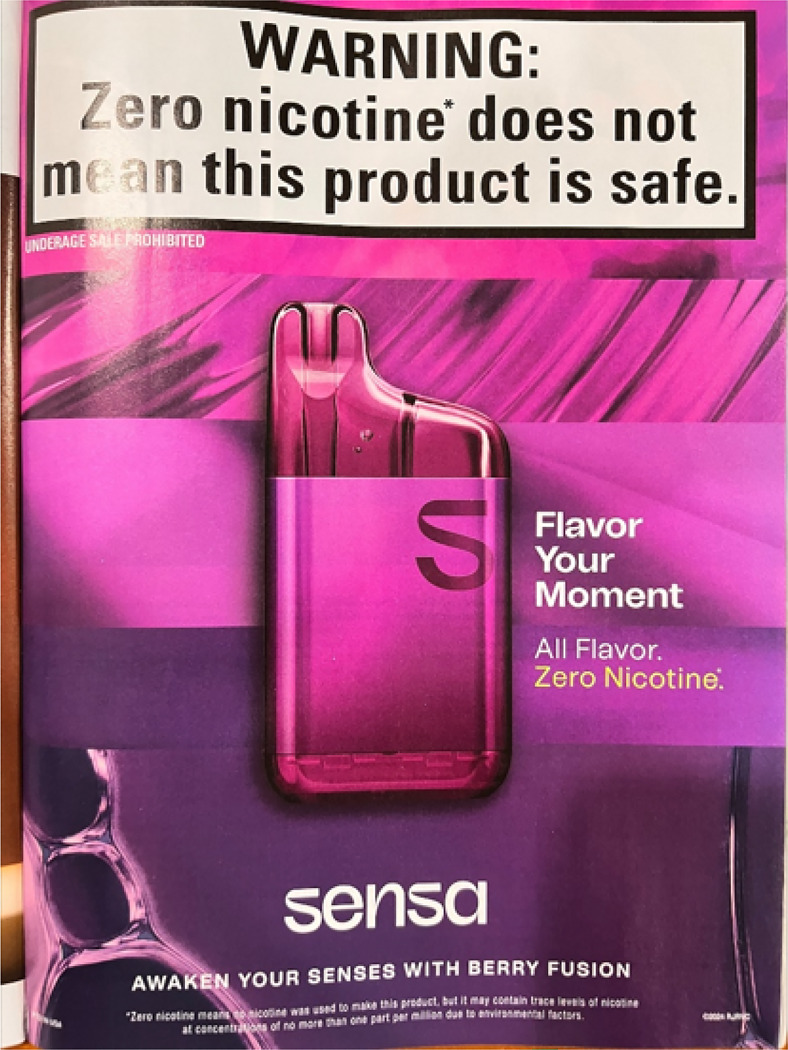
Sensa ad in the September 2024 issue of *Bon Appétit* magazine (a food lifestyle magazine in the U.S.)

**Figure 3:**
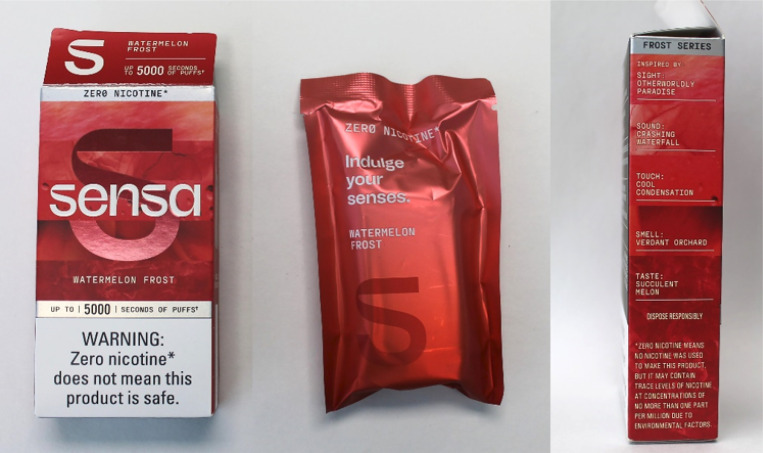
Outer and inner packaging for Sensa’s Watermelon Frost flavor (purchased in Maryland on August 20, 2024)

Sensa launched in 22 states and has been promoted through several channels. ^6^ Direct-mail ads sent July 2024 included $7–$10 coupons and links to additional offers through the age-gated brand website (Fig. 1). The ad came in a shiny purple foil envelope bearing the brand logo and a warning label (“WARNING: Zero nicotine does not mean this product is safe”). The inner mailer described Sensa as “user-friendly, flavor forward” and listed device features, including transparent casing to view “liquid levels,” a locking safety switch, and a rechargeable, removeable battery for “responsible disposal.” Sensa’s six flavor options each contain fruit and/or ice-adjacent descriptors—Berry Fusion, Berry Watermelon Fusion, Blueberry Frost, Mint Frost, Passionfruit Frost, and Watermelon Frost. An ad in the September 2024 issue of *Bon Appétit* magazine highlighted the Berry Fusion flavor, with the copy, “Flavor Your Moment” (Fig. 2). The product packaging also appeals to consumers’ senses by describing sights, sounds, tastes, smells, and tactile stimuli that inspired each flavor (Fig. 3). Sensa’s website claims that their products contain “food-grade flavorings designed for an adult palate”—a misleading claim since food safety determinations do not extend to inhalation safety. ^5,7^

## Materials & Methods

We purchased all Sensa flavors (except Berry Watermelon Fusion) from North Carolina in July, 2024 for $18.99 each (before tax or coupons) and conducted chemical characterization using established methods. ^8^ Briefly, e-liquid samples were extracted from the device, diluted in methanol containing 1,4-dioxane as internal standard, and characterized using gas chromatography coupled with mass spectroscopy (GC-MS: PerkinElmer Clarus 580-SQ8S fitted with an Elite-5MS column: length 60 m, id 0.25 mm, 0.25 μm film), and WS-3 and WS-23 were quantified using commercial analytical standards using GC coupled with a flame ionization detector (GC-FID: Shimadzu GC-2010 Plus fitted with an Agilent J&W DB-5 column: length 60 m, id 0.25 mm, 0.25 μm film). In addition, samples diluted 1000-fold with methanol were analyzed for sweetener presence using liquid chromatography coupled with MS (GC/MS/MS: Thermo Quantis fitted with a Thermo Accucore RP-MS column: length 50mm, diameter 2.1mm, particle size 2.6 μm) using acetonitrile and water as mobile phases.

## Results

Chemical analysis (Table 1) showed that neither nicotine nor the nicotine analogs 6-methylnicotine or nicotinamide were detected in any sample. However, all samples contained neotame (range: 1.2–mg/g)—an artificial sweetener 7,000–13,000 times sweeter than sugar—which has recently been found in other disposable devices.^8,9^ Additionally, all samples contained the synthetic coolant WS-23 (range: 12.2–36.3mg/g), and all “Frost” flavors tested contained another synthetic cooling agent, WS-3 (range: 7.8–8.0mg/g). These amounts are comparable to those reported for some nicotine-containing disposable devices.^10–12^

## Discussion

RJRVC states that Sensa is for “adult tobacco and vapor consumers,” claiming it will treat Sensa as a nicotine product for marketing and distribution purposes.^1^ However, Sensa’s resemblance to popular disposable vaping devices and the presence of youth-appealing flavors, sweeteners, and coolants raise concerns about Sensa’s appeal to young people. The long-term health effects of these chemical constituents remain unclear, and limited regulations for “zero nicotine” vaping devices introduce uncertainty about product safety.^10,12,13^ Additionally, commercial success of Sensa could further normalize vaping and increase concerns over environmental impacts of disposables.

Although “zero nicotine” products constitute a small percentage of the e-cigarette market share, Nielsen retail scanner data through September 10, 2024 indicates that Sensa sold over 100,000 units since launching in July, suggesting consumer interest.^14^ Given potential concerns about product safety and youth appeal, Sensa and similar “zero nicotine” disposable vaping devices should be monitored closely.

## Figures and Tables

**Table 1:** Chemical Characterization of Five Sensa Flavors for the Sweetener Neotame, the Two Synthetic Coolants WS-3 and WS-23, and other major detected flavorants.

Sensa Flavor	PG/GL* ratio	Neotame (mg/g) (mean, SD)	WS-23 (mg/g) (mean, SD)	WS-3 (mg/g) (mean, SD)	Detected major flavorants^†^
Berry Fusion	55/45	3.0 (0.2)	12.2 (0.2)	n.d.	Benzyl alcohol, vanillin, ethylvanillin, ethyl maltol, maltol
Blueberry Frost	60/40	1.6 (0.2)	36.3 (0.1)	7.9 (0.1)	Benzyl alcohol, vanillin, ethylvanillin, ethyl maltol, piperonal, ethyl butanoate, other fruity esters
Mint Frost	60/40	1.2 (0.3)	35.0 (1.6)	7.8 (0.3)	Benzyl alcohol, menthol, menthone, carvone, vanillin, ethylvanillin
Passionfruit Frost	60/40	1.8 (0.1)	18.9 (0.8)	7.9 (0.2)	Benzyl alcohol, vanillin, ethylvanillin, fruity esters
Watermelon Frost	65/35	2.1 (0.3)	19.9 (0.3)	8.0 (0.1)	Benzyl alcohol, vanillin, linalool, creosol, geraniol, gamma-decalactone

* PG: Propylene glycol, GL: glycerol

† Not a comprehensive list of flavorants
